# Predicting gastrointestinal drug effects using contextualized metabolic models

**DOI:** 10.1371/journal.pcbi.1007100

**Published:** 2019-06-26

**Authors:** Marouen Ben Guebila, Ines Thiele

**Affiliations:** 1 Luxembourg Centre for Systems Biomedicine, University of Luxembourg, Esch-sur-Alzette, Luxembourg; 2 School of Medicine, National University of Ireland, Galway, University Road, Galway, Ireland; 3 Discipline of Microbiology, School of Natural Sciences, National University of Ireland, Galway, University Road, Galway, Ireland; University of Toronto, CANADA

## Abstract

Gastrointestinal side effects are among the most common classes of adverse reactions associated with orally absorbed drugs. These effects decrease patient compliance with the treatment and induce undesirable physiological effects. The prediction of drug action on the gut wall based on *in vitro* data solely can improve the safety of marketed drugs and first-in-human trials of new chemical entities. We used publicly available data of drug-induced gene expression changes to build drug-specific small intestine epithelial cell metabolic models. The combination of measured *in vitro* gene expression and *in silico* predicted metabolic rates in the gut wall was used as features for a multilabel support vector machine to predict the occurrence of side effects. We showed that combining local gut wall-specific metabolism with gene expression performs better than gene expression alone, which indicates the role of small intestine metabolism in the development of adverse reactions. Furthermore, we reclassified FDA-labeled drugs with respect to their genetic and metabolic profiles to show hidden similarities between seemingly different drugs. The linkage of xenobiotics to their transcriptomic and metabolic profiles could take pharmacology far beyond the usual indication-based classifications.

## Author summary

The gut wall is the first barrier that encounters orally absorbed drugs, and it substantially modulates the bioavailability of drugs and supports several classes of side effects. We developed context-specific metabolic models of the enterocyte constrained by drug-induced gene expression and trained a machine learning classifier using metabolic reaction rates as features to predict the occurrence of side effects. Additionally, we clustered the compounds based on their metabolic and transcriptomic features to find similarities between their physiological effects. Our work provides a better understanding of the compound physiological effects solely using *in vitro* data, which can further improve the translation of new chemical entities to clinical trials.

## Introduction

Side effects are unintended effects of administered drugs that lead to a decrease in the efficacy of treatment, lower patient compliance, and eventually the cessation of treatment with the development of adverse physiological consequences. Additionally, up to 25% of drug development programs fail because of a lack of safety in first-in-human trials [1]. In particular, since the oral administration of drugs is the most common route of disposition, the gastrointestinal side effects are among the most common class by occurrence [2, 3], particularly in geriatrics [4]. Therefore, identifying compounds that can cause serious gastrointestinal adverse reactions from the ones that have benign effects solely using *in vitro* data could help optimizing drugs in the preclinical phase before first-in-human trials and ultimately, decrease the failure rates of new chemical entities.

The prediction of side effects have been addressed mainly through a target-based approach wherein the inhibition of a specific target induces the desired effect and also suppresses all physiological processes involving the target protein [5]. Recently, with the availability of genome-wide transcriptome profiles of more than 20,000 compounds in the connectivity map [6], new approaches have considered linking off-target effects rather than target effects to adverse reactions. Specifically, the interaction of the compound with nontarget genes has been hypothesized to drive the emergence of side effects [7]. Recent efforts have combined drug-induced gene expression with chemical structures and Gene Ontology (GO) processes as features to predict side effects accurately [8]. Notably, metabolic genes are among the most predictive features for the classification [8].

Additionally, context-specific drug metabolic models have been built using a generic genome-scale reconstruction of human metabolism [9] and the connectivity map of gene expression [6] to identify metabolic dysregulation underlying the emergence of side effects [10].

**Fig 1:**
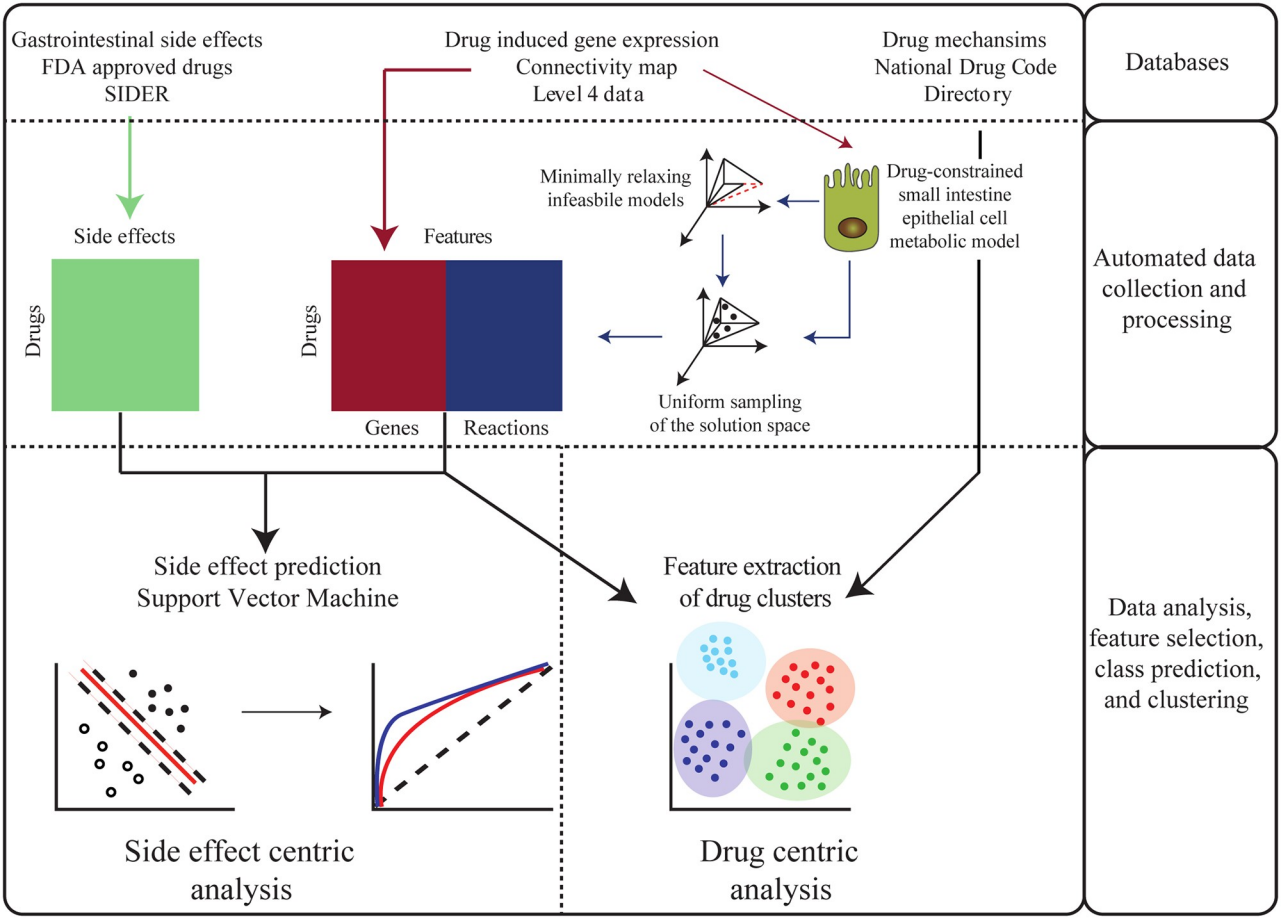
Overview of the pipeline of data generation and analysis in this study. Drug-induced gene expression data was collected through the connectivity map API for 978 genes [6]. The drug side effects occurrence was obtained from the SIDER database, and the drug mechanism and physiological effects were collected from the FDA National Drug Code Directory (NDCD). The drug-induced gene expression was subjected as constraints to an sIEC metabolic model to derive a context-specific model for each drug, to enable the prediction of the enterocyte’s metabolic phenotype after the action of the drug. After minimally resolving infeasible models, the uniform sampling of the solution space of the sIEC provides distributions of fluxes for each reaction and could be used to derive z-scores for the 1282 metabolic reactions in a given drug-constrained model and the drug-free model. Combining the drug-induced gene expression with predicted differential metabolic fluxes in a single matrix was used to train and cross-validate a multilabel support vector machine, using the SIDER side effect labels as the class binary matrix (M), where if drug *j* causes side effect *i*, *M*(*i*, *j*) = 1, and 0 otherwise. Such classifier can be used to predict the likelihood of occurrence of side effects of newly developed drugs based on their *in vitro* transcriptomic fingerprint and their *in silico* predicted metabolic profile in the enterocyte. Finally, the clustering of drugs based on their transcriptomic and metabolic profiles can be used to identify hidden similarities between the compounds that go beyond the chemical or pharmacological class-based classification.

In this study, we first considered a metabolic model of the small intestine epithelial cells (sIECs), where drug-induced gene expression data constrained the set of predicted metabolic phenotypes (Fig 1). The assessment of possible reaction rates, while constrained by gene expression data, was used to derive differential scores between the drug-specific model and the unperturbed model. Second, we used the corresponding metabolic reactions and the drug-induced gene expression taken as features to build and cross-validate a multilabel support vector machine in order to predict the occurrence of gastrointestinal side effects. Such a classifier could be applied to drugs in preclinical development based on *in vitro* parameters solely to predict the likelihood of side effect occurrence in first-in-human trials. Finally, the transcriptomic and metabolic profiles of drugs were used to cluster compounds by their signatures, enabling a new classification that goes beyond the usual chemical class, thereby offering new insights into drug repurposing.

The combination of local sIEC metabolism [11] with drug transcriptomic profile allowed to contextualize gene expression data thereby increasing the predictive capability of side effect classifiers. Extending the classification to a more comprehensive set of side effect and tissue-specific models could provide useful information at the preclinical phase of drug development thus reducing costs and attrition rates.

## Materials and methods

### Small intestine epithelial cell model

A manually curated metabolic model of sIECs has been previously constructed to study the effect of inborn errors of metabolism (IEMs) on human physiology [11]. The sIEC model consists of 1282 reactions and 844 metabolites. The exchange reactions for the sIEC model has been set for a standard European diet, as described previously [11], over an interval of 24 h. Consequently, we prioritized drug-induced gene expression measured after 24 h on intestinal cell lines, namely, HT115, MDST8, SW-948, NCI-H716, HT-29, SW620, HCT 116, and LoVo obtained from the LINCS database [6, 12] (see ‘Data generation’). For each drug, an sIEC-tailored metabolic model was generated in the form of a linear program (LP) as follows:

$$\begin{array}{ll} \text{max}: & c^{T}v\\ \text{subject}\;\text{to}:\; & \\ & Sv=0\\ & v_{min}\leq v\leq v_{max} \end{array}$$ (1)

where *c*^*T*^.*v* is the objective function, *v* is the flux vector of metabolic reactions, *c* is the vector of objective coefficients, *S*_(*m*, *n*)_ is the stoichiometric matrix linking *m* metabolites and *n* reactions, *v*_*min*_ is the reaction lower bound vector, and *v*_*max*_ is the reaction upper bound vector. The system assumes a steady state such that *S*.*v* = 0, which is referred to as flux balance analysis (FBA) [13].

### Subjecting gene expression as constraints on metabolic models

Differential gene expression *z*_*i*_ of gene *i* encoding reaction *j* modifies the allowable range of each reaction obtained by flux variability analysis (FVA) [14], which determines the minimal and maximal values feasible by each reaction, through maximizing and minimizing each reaction as an objective function, consistent with the applied constraints. The constraints were updated as follows:

$$\begin{array}{ll} v_{min,j} & =min_{FVA,j}+z_{i}*std(v_{j})\\ v_{max,j} & =max_{FVA,j}+z_{i}*std(v_{j}) \end{array}$$

where *v*_*min*_ and *v*_*max*_ are the new lower and upper bounds of the sIEC drug model respectively; *min*_*FVA*_ and *max*_*FVA*_ are the lower and upper bounds of the drug-free sIEC determined by FVA, respectively; and *std*(*v*_*j*_) is the standard deviation in reaction *j* assuming a normal distribution of the fluxes between *min*_*FVA*_ and *max*_*FVA*_. This formulation of reaction constraints is similar to E-Flux [15, 16] and retains the original structure of the model while changing the reaction bounds according to the gene expression. This formulation was chosen because transcript levels cannot be used as conclusive evidence of the enzymatic activity of proteins [17–19] and metabolic fluxes but are rather used to constrain the capacity and space of possible flux values of the corresponding reaction. Because FVA-calculated minimal and maximal bounds determine the solution space, scaling FVA bounds by gene expression constrains a new space of predicted phenotypes. Other recent formulations have considered protein concentrations to constrain flux capacities [20].

An infeasible sIEC-drug model may occur because of conflicting constraints, particularly with the exchange reactions. If problem 1 was infeasible, then we minimally relaxed the constraints in both the amplitude of relaxation and the cardinal of relaxed reactions [21], by solving the following problem:

$$\begin{array}{ll} \text{min}:\; & {\left|\left|p\right|\right|}_{1},{\left|\left|q\right|\right|}_{1}\\ \text{subject}\;\text{to}:\; & \\ & Sv=0\\ & v_{min}-p\leq v\leq v_{max}+q \end{array}$$

where *p* is the relaxation vector of the lower bound and *q* is the relaxation vector of the upper bound. Minimizing the 1-norm of *p* and *q* ensures sparsity (a minimal cardinal of reactions to be relaxed) and a minimal total sum of relaxation amplitudes [21].

Under the drug constraints, we calculated the possible flux values for each of the 1282 reactions through the uniform sampling of the LP solution space using Artificially Centered Hit-and-Run (ACHR) implemented in the COBRA Toolbox [21]. Sampling is an unbiased method because it does not assume any objective function. We generated 100,000 points for each model using 1000 iteration steps per point, starting from 10,000 warmup points. The sampling of metabolic models has been used to determine a set of phenotypes of the modeled condition-specific cells and the distribution of reaction rates under a set of applied constraints [22–24]. For each metabolic reaction of a specific drug-constrained sIEC, the sampled flux distribution was compared to the drug-free sIEC model, and z-scores were derived for each reaction.

### Data generation

We used the Side Effect Resource (SIDER) [5, 25] side effect database to extract the intestinal side effects described as preferred terms (PTs). The compounds corresponding to a side effect were then queried in the L1000 LINCS dataset of compound gene expression [6, 12] through the iLINCS API [26]. The level four data reported differential expression z-scores of the 978 measured genes [6]. On average, 50 drug gene expression signature genes overlapped with the genes present in the sIEC model [11], and only the genes that were differentially expressed with a p-value lower than 0.05 were retained for further analyses. These genes were used for setting the constraints, as aforementioned.

We created a feature matrix consisting of gene expression and metabolic flux samples in columns and drugs in rows representing the observations. The matrix had 605 drugs and 2260 features (978 genes plus 1282 metabolic reactions). Standardized predictors were directly used as z-scores for learning and cross-validation. We calculated the minimal and maximal flux capacity for each reaction using FVA. The resulting flux values served as features in the classification, as previously suggested [27]. This second feature matrix had 605 drugs and 1282*2 columns. We also considered the gene expression data alone, yielding a third feature matrix consisting of 605 drugs and 978 genes.

### Building a multilabel support vector machine

The support vector machine multilabel learning was converted to 43 binary single-label problems using binary relevance in a one-versus-all scheme, where each classifier corresponded to an intestinal side effect as reported by SIDER PTs. The side effects occurring for only one drug were discarded, resulting the final set to 36 side effects. The dataset used in classification was standardized in the SVM call, where the mean is subtracted from each entry, followed by a division by the standard deviation of the training set.

The support vector machine classifier [28] was compared to random forest [29], logistic regression [30], and Naïve Bayes [31] with their defaults parameters (S1 Fig). The performance was assessed using the following metrics [32]: accuracy, area under the ROC curve (AUROC), area under the precision-recall curve (AUPR), weighted accuracy, and weighted recall. The weighted recall and weighted accuracy were calculated using the average of the accuracy and recall of each label and weighted by the label size. The significance of the difference between the AUROC of the classifiers was determined using the Hanley and McNeil test [33], which is a nonparametric method that corrects for the correlation between ROC curves derived from the same cases i.g., drugs.

The genes and metabolic reactions were then ranked by importance and used as input for the SVM multilabel model.

#### Feature selection algorithm

Given the high number of features, we proceeded to the selection of the most predictive genes and metabolic reactions using the feature selection toolbox [34] implemented in MATLAB (2017a release, Natick, MA, USA). We tested 11 methods of feature selection and compared them with regard to the performance of the SVM classifier as assessed by the AUROC (S2 Fig). The algorithms tested with their default parameters were ReliefF [35], mutinffs [36], FSV [37], Laplacian [38], MCFS [39], L0 [40], Fisher score [41], udfs [42], llcfs, and cfs [43]. ReliefF showed the highest predictive capability for the selected features and hence, was used for feature selection. Briefly, the algorithm ranks the features by importance based on a k-nearest neighbor graph. Consequently, the k parameter had to be optimized.

#### k parameter of ReliefF

The k parameter of ReliefF was varied through a range of values, and the results were compared with respect to the AUROC. Usually, a low value of k could not be used to generate a strong separation of predictive features, whereas a k value equal to the number of drugs would lead to the failure of the algorithm. A k value of 80 yielded the highest AUROC (S3 Fig).

#### Number of features

The number of predictive features was assessed by testing different values. The ReliefF algorithm takes as input the feature matrix and the corresponding side effect labels of the training set and calculates the ranking and weights of the features. Selecting 20 features yielded the highest AUROC (S4 Fig).

#### Cross-validation method

To avoid over-fitting and to enhance the accuracy of the classifier, two cross-validation methods were tested. K-fold cross-validation consists of splitting the dataset into k parts and performing learning on k-1 folds, and the last fold is used for testing. The leave-one-out cross-validation trains the classifier on n-1 points and validates the prediction on the n^th^ data point. The 3-, 5-, and 10-fold cross-validation, as well as leave-one-out, were compared for loss and predictability (S5 Fig). Because the cross-validation methods seemed comparable in prediction outcomes (S5 Fig), we selected 3-fold cross-validation for further analysis because it requires less computational time. The process is summarized in the following steps:

1. Divide the dataset into test (20%) and training (80%) set;
2. Train a single-label classifier for each label with 3-fold cross-validation on the training set;
3. Repeat step two twice with a different partition of the training and validation set;
4. Predict the label of the test set using the trained models;
5. Repeat step one to four 100 times, each time taking a different partition of the test and training set.

Finally, the posterior probabilities and the prediction loss on the test set were averaged for each side effect label.

#### Misclassification cost

Class imbalance is frequently encountered in biological datasets [44, 45]. In our case, the occurrence of intestinal side effects varies widely from frequent unspecific disorders to rare side effects occurring with a few drugs. The misclassification cost matrix *C* was set to the inverse of the label frequencies such that:

$$C=\left(\begin{array}{cc} 0 & \frac{1}{n_{t}-S_{f}}\\ \frac{1}{S_{f}} & 0 \end{array}\right)$$

where the rows correspond to the observed labels, the columns correspond to the predicted labels in each binary classifier, *n*_*t*_ is the size of the training set, and *S*_*f*_ the side effect frequency. The effect of class balance improved the classification performance (S6 Fig).

#### Observation weight

For every drug, intestinal side effects occur with different empirical frequencies in a population of patients, as reported by post-marketing adverse reaction reporting. For every label, the weight of every observation, i.e., drug, in the classifier was set to its empirical frequency as reported in the SIDER database, in such a way to select the features of the drugs that most commonly induce the side effect. Information on 485 side effect frequencies was available over a total number of 1053 side effects induced in the 36 labels. When no information was available, we set the frequency to one, without performing additional data imputation. Adding observation weights induced a slight decrease in the mean of the AUROC for intestinal side effects (S7 Fig) and was not subsequently kept as a parameter in the model, which was likely because of the effect of the missing 54% of side effect frequencies.

#### SVM kernel

The kernel functions of support vector machines tested included a linear, a Gaussian, and a 3^rd^ order polynomial function. The Gaussian kernel function had the highest mean of the AUROC per label (S8 Fig) and was consequently set as a label-wide function. We also identified the optimal kernel functions per label using a MATLAB hyperparameter optimization routine *OptimizeHyperparameters*.

#### Optimal hyperparameters

The optimal hyperparameters obtained for the SVM in previous analyses and summarized in S1 Table ensured high predictability and accuracy. Additionally, the automatic tuning of hyperparameters available in MATLAB was tested for hyperparameters listed in S2 Table. The automatic tuning routine finds the optimal set of parameters that minimize the cross-validation loss, where each binary classifier can have a different set of optimal parameters. Manually tuning the global hyperparameters produced a better AUROC curve than individually optimized hyperparameters (S9 Fig) because the set of automatically tuned parameters in the 2017a release of MATLAB does not include all of the parameters, particularly “number of features” and “feature selection method”.

### Drug community identification, validation, and interpretation

#### Graph clustering

A significance test on the principal components was performed using 100 independent permutations of the columns of the feature matrix [46]. Fifteen principal components had a p-value of < 0.001 and were retained for subsequent analysis. Then, we constructed a graph based on the k-nearest-neighbor (KNN) [47] of each drug with k equal to 20, using the Jaccard index as a distance metric.

$$\begin{array}{c} J\left(d_{A},d_{B}\right)=\frac{|d_{A}\cap d_{B}|}{|d_{A}\cup d_{B}|} \end{array}$$

where *d*_*A*_ and *d*_*B*_ are two given drugs in the networks, |*d*_*A*_ ∩ *d*_*B*_| is the number of common neighbors and |*d*_*A*_ ∪ *d*_*B*_| is the union of drug neighbors.

#### Community detection

Drug clusters in the network were identified using the Jaccard-Louvain algorithm [48] as previously reported [49] and the second level of community clustering produced eight clusters and was selected for further analysis. The Jaccard-Louvain algorithm is based on two-steps. First, it assigns drugs to communities such that local modularity is optimized. In a second step, it aggregates local communities to build a coarse-grained network. The process is repeated until no improvement in modularity is observed.

#### Cluster visualization

For further validation and visual inspection, the clusters were visualized using Barnes-Hut Stochastic Neighborhood Embedding (Bh-SNE) [50], with Euclidean distance, a perplexity of 30 and exaggeration of four on the 15 first principal components of the combined feature matrix. The seed number used for the plot was 97.

#### Cluster validation

We performed network perturbations to assess the stability of the identified clusters. The value of k in the KNN algorithm was randomly selected in a uniform distribution between two and 50. We then independently selected 85% of the drugs and built the Jaccard-based adjacency matrix, of which we removed 5% of the edges and added 5% of new random edges. Finally, random noise values were added to the edges and the Jaccard-Louvain [48] algorithm identified communities in the perturbed network. The process was repeated 200 times. To validate the selected clusters, stability and purity were selected as external measures and were calculated for each cluster [51] as previously reported [49]. Stability is a measure of diversity in the clusters of each perturbation trial. Briefly, if the points assigned to a given cluster in a given trial have a high diversity of points from the original clustering assignment, the stability would be low. Stability was calculated as follows:

$$\begin{array}{c} \left\{ \begin{array}{c} Stability=1-instability\\ Instability=\frac{1}{n}\sum_{i=1}^{n}\frac{E_{i}}{E_{i}^{Tot}}\\ E_{i}=\sum_{i}p_{i}log\left(p_{i}\right)\\ E_{i}^{Tot}=\sum_{j=1}^{m}p_{j}E_{j} \end{array}\right. \end{array}$$

where *E*_*i*_ is the entropy of a given cluster in trial *i*, $E_{i}^{Tot}$ is the total entropy in trial *i*, *p*_*i*_ is the fraction of a given cluster size over all data points in trial *i*, *m* is the number of clusters, and *n* is the number of trials. Purity consists of the enumeration of data points in a given cluster *i* that were labeled as cluster *j*. Purity is calculated as follows:

$$\begin{array}{c} \left\{ \begin{array}{c} Purity=1-\sum_{j=1}^{m}\frac{|cl_{j}|}{\left|nDrugs\right|}P_{j}\\ P_{j}=\frac{1}{|cl_{j}|}Max_{i}(|cl_{j}^{i}\left|\right) \end{array}\right. \end{array}$$

where *m* is the number of clusters, |*cl*_*j*_| is the size of cluster *j*, *P*_*j*_ is the purity of cluster *j*, and $Max_{i}(|cl_{j}^{i}\left|\right)$ is the size of the largest cluster *i* contained in cluster *j* of the unperturbed clustering.

#### Graph representation

In order to visualize common patterns in the identified clusters, we built a bipartite graph linking the compounds identified in each cluster to their mechanism of action, their physiological effect, and their established pharmacological class as reported by the Food and Drug Administration (FDA) National Drug Code Directory [52]. The diagram was drawn using Rawgraphs [53].

#### Gene set enrichment

For each cluster, the top differentially expressed genes were identified and submitted for pathway enrichment in KEGG [54] via the Enrichr API [55, 56]. Then, the ten most significantly enriched terms (*p* < 0.05) were assigned to each cluster and linked to the corresponding drugs via a bipartite graph as described above.

## Results

The prediction of iatrogenic gastrointestinal drug effects based on *in vitro* data could improve the assessment of the safety of new chemical entities in early phases of drug development. We combined *in vitro* drug-induced gene expression data with *in silico* metabolic models of the sIEC as features to develop a machine learning classifier of side effects. We employed the classifier to predict the occurrence of common gastrointestinal side effects taken from the SIDER database [57] and the biological processes involved in their development. In particular, we showed that adding sIEC metabolic reactions as features to the gene expression data in the side effect classifier could capture local gut metabolism and improve the accuracy of the classifier. Furthermore, we clustered the drugs using their obtained transcriptomic and metabolic signatures to suggest similarities into drug action and mechanisms and to provide drug repurposing hypotheses. Finally, including more tissues through context-specific metabolic models could extend the approach to additional labels of side effects.

### Combining measured gene expression and simulated gut wall metabolism predicts iatrogenic intestinal effects

**Fig 2:**
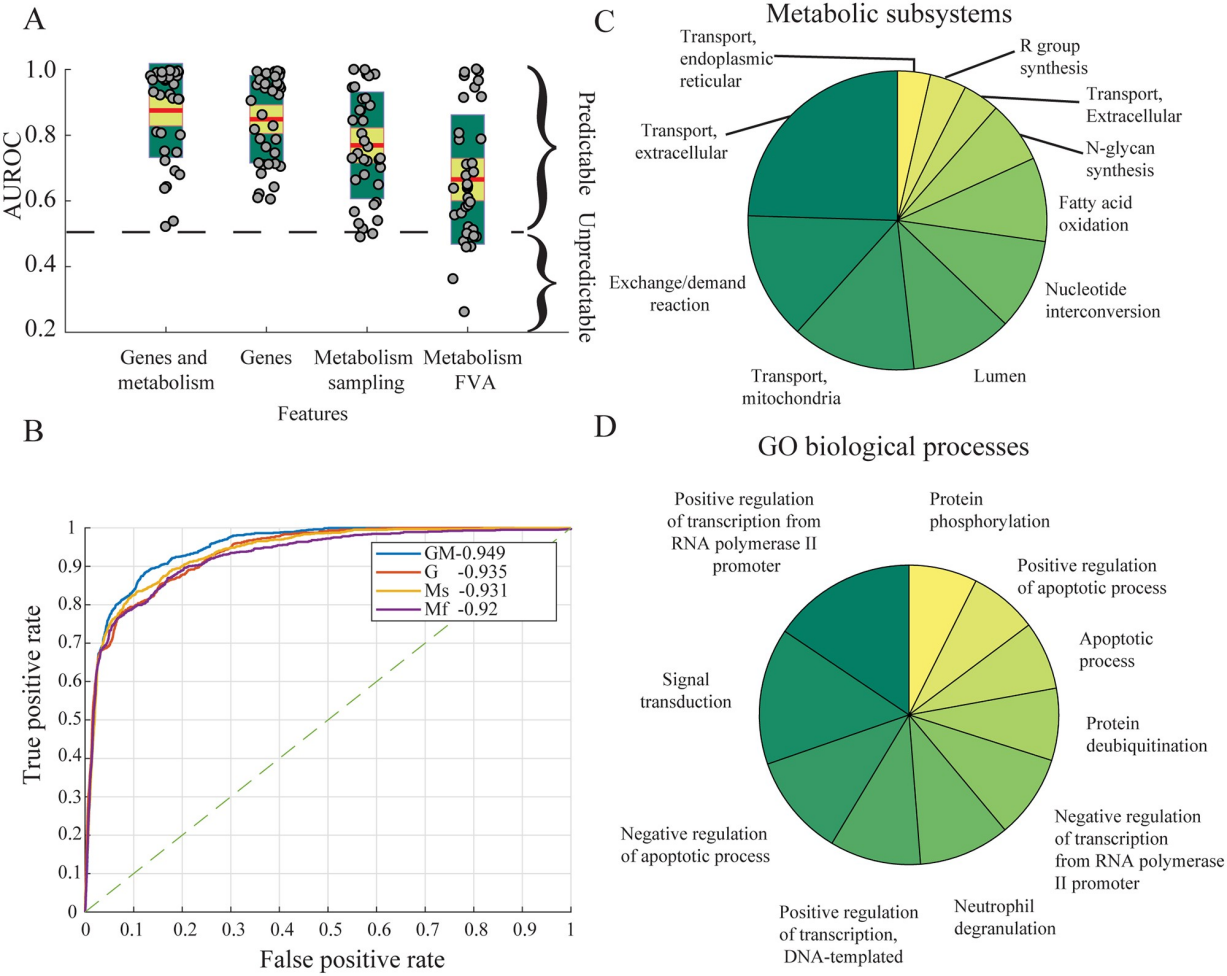
Evaluation metrics for the multilabel support vector machine intestinal side effects classifier. **A**—Comparison of individual side effect classifier predictive capability as measured by the out-of-sample AUROC using genetic, metabolic, and combined genetic and metabolic features with a 95% confidence interval around the mean in yellow and one standard deviation in green. **B**—ROC curve of the microaveraged multilabel SVM classifier using genes, metabolism and combined genes and metabolism as features. All the pairwise differences between the AUROC are significant at p <0.05 using the Hanley and McNeil test [33], except the difference between the classifier using sampled reactions as features and the classifier using gene expression as features. Unlike panel **A** that summarizes the AUROC curves where each class is treated equally, the micro-average allows to correct for the class imbalance. **C**—Top ten enriched metabolic subsystems and **D**—GO biological process terms ranked by the number of metabolic and genetic representatives. G stands for genes, Ms for metabolism sampling, Mf for metabolism FVA, and GMs for genes and metabolism sampling.

Drug-induced gene expression can provide strong features for side effect prediction, especially with side effect labels that have links to metabolism [8]. We used this observation to further improve the prediction of intestinal side effects by subjecting gene expression as constraints in the metabolic model of the sIEC, thereby contextualizing sIEC metabolism to derive the intestinal metabolic fingerprints of every drug. This approach combines features linked to side effects in both the gene expression space and the metabolism of the entercoyte. Consistent with a previous study [8], the gene expression data alone was the support of most predictive features (Fig 2-A). Metabolic reaction fluxes alone were less predictive than gene expression as they only consider genes with defined metabolic activity in the sIEC model (Fig 2-A). Particularly, sampling the metabolic model improved the classification because it provided information about the distribution of metabolic fluxes for each reaction rather than only having the minimal and maximal flux values when using FVA (Fig 2-A). Combining gene expression and predicted metabolism gave the highest predictive rates in the multilabel SVM classifier as the average of individual labels (Fig 2-A), and in comparison to other classifiers (S1 Fig).

The merged multilabel intestinal side effect classifier using the individual binary classifiers of each side effect better predicted (AUROC = 0.94) intestinal side effects based on the combination of gene expression and metabolic flux distributions compared to 1. gene expression alone (AUROC = 0.935, p = 0.02), 2. FVA predictions alone (AUROC = 0.92, p = 0.00005), or 3. sampling results alone (AUROC = 0.931, p = 0.006) as shown by the microaveraged ROC curve (Fig 2-B).

**Fig 3:**
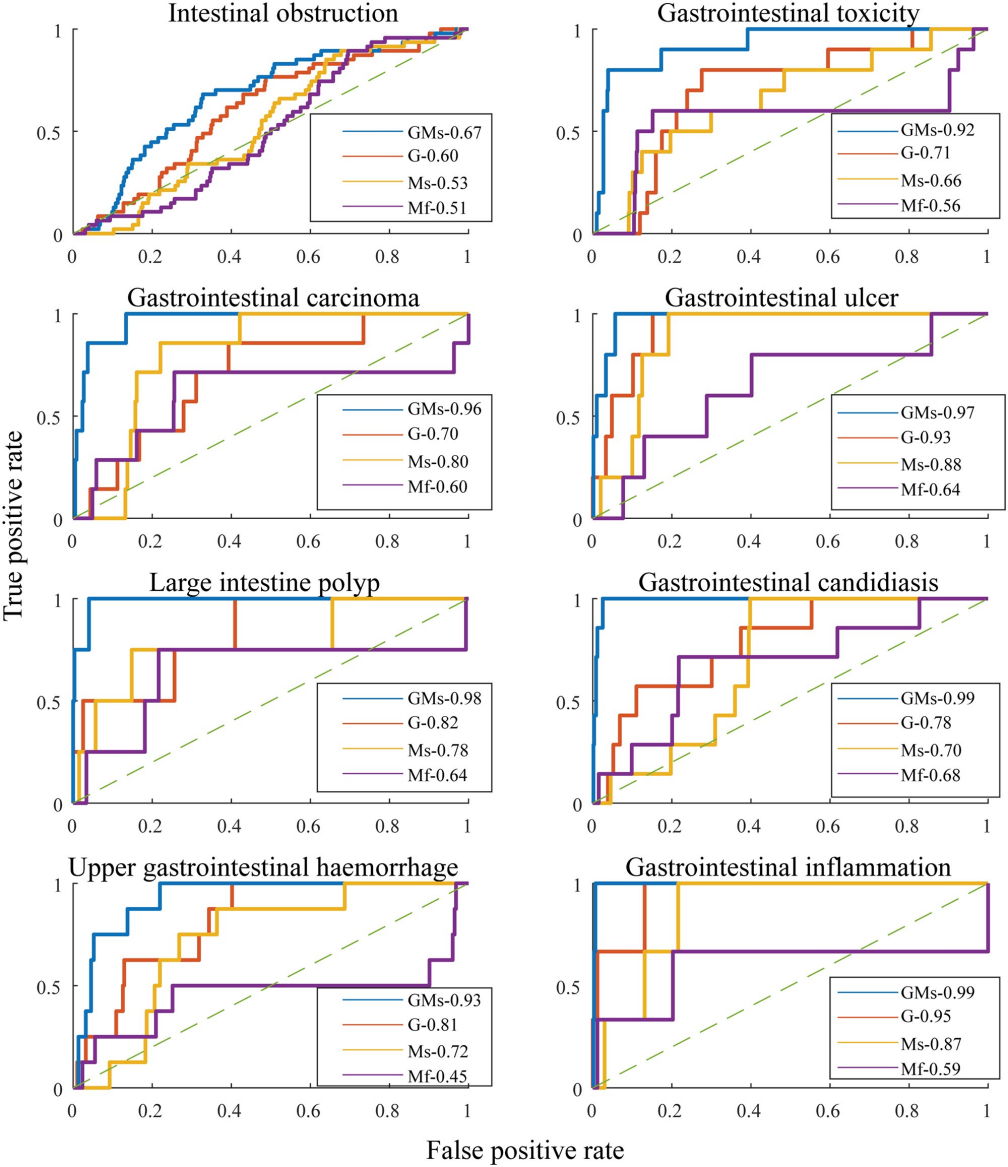
Out-of-sample ROC curve for intestinal obstruction, gastrointestinal toxicity, carcinoma, ulcer, polyp, candidiasis, hemorrhage, and inflammation. The features used for comparison were the sampled flux values in metabolic models, the minimal and maximal metabolic flux values per reaction as determined using FVA, gene expression as reported in the connectivity map, and the combined gene expression and sampled reaction flux values. The comparison was performed using the AUROC of each classifier. The full list of side effects and the corresponding AUROC values can be found in S3 Table. G stands for genes, Ms stands for metabolism sampling, Mf stands for metabolism FVA, and GMs stands for genes combined with metabolism sampling.

The most predictive metabolic reactions were enriched in the subsystems of sIEC, and the most predictive genetic features were enriched in the GO biological processes database. The ten most represented subsystems (p <0.001) mainly involved transport reactions (extracellular, exchange, mitochondrial, and endoplasmic reticulum) as well as catabolic and anabolic functionalities (Fig 2-C). The GO biological processes-enriched groups (p <0.001) involved mainly the regulation of transcription and apoptotic processes (Fig 2-D). Unspecific or likely nonmetabolic side effects, such as gastrointestinal obstruction, were among the least predictable with an AUROC of 0.67 using combined gene expression and sampled reaction fluxes. Side effects involving the gut wall metabolism were highly predictable using combined features (Fig 3, S3 Table), such as intestinal carcinoma (0.96), ulcer (0.97), and toxicity (0.92).

These results motivated us to employed in the following the matrix of combined drug transcriptomic and metabolic features to predict the labels of gastrointestinal side effects. Therefore, we used this matrix to perform a drug-centric analysis in order to cluster drugs with respect to their metabolic and transcriptomic signatures and investigate common genome-scale similarities of drug action that can provide new drug repurposing strategies.

### Drug classification using transcriptional and intestinal metabolic activity

**Fig 4:**
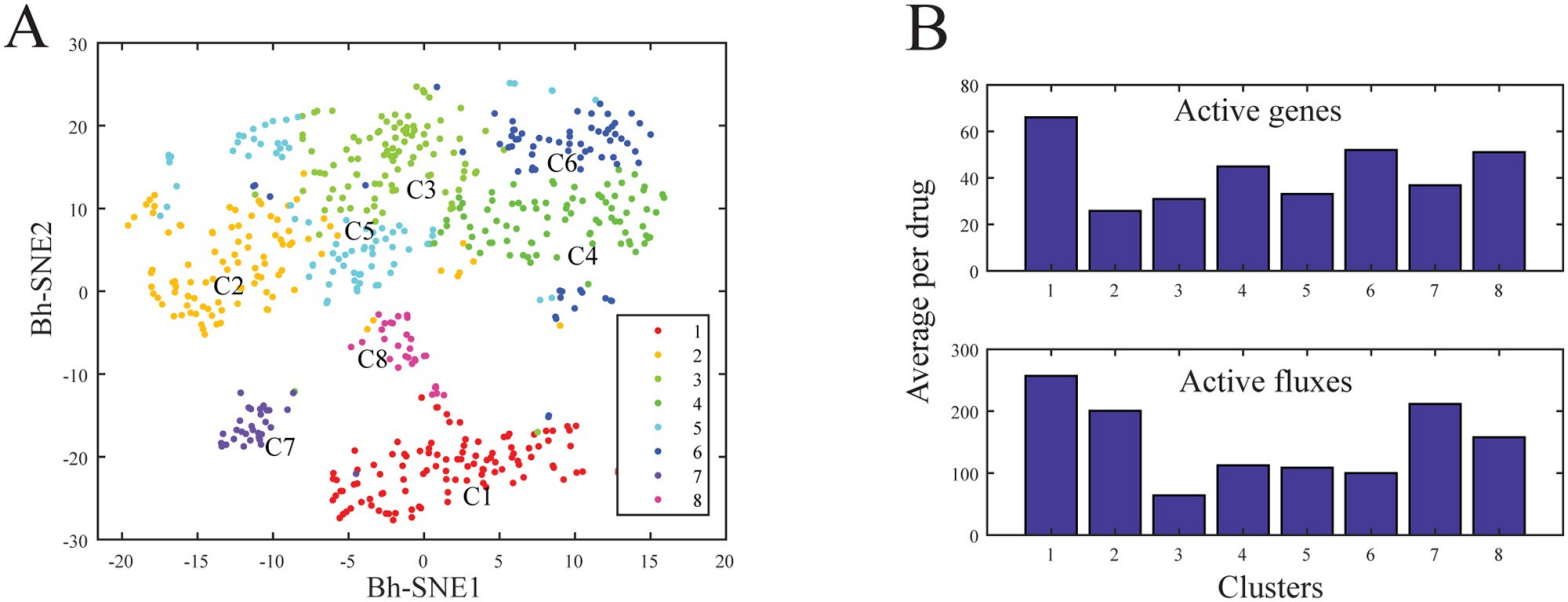
Drug community identification based on measured transcriptional and simulated gut wall metabolic profile. A-Visualization of the eight validated drug clusters through a Bh-SNE plot. B-Transcriptional and gut metabolic activity of the identified clusters showed different levels of drug specificity per cluster.

**Fig 5:**
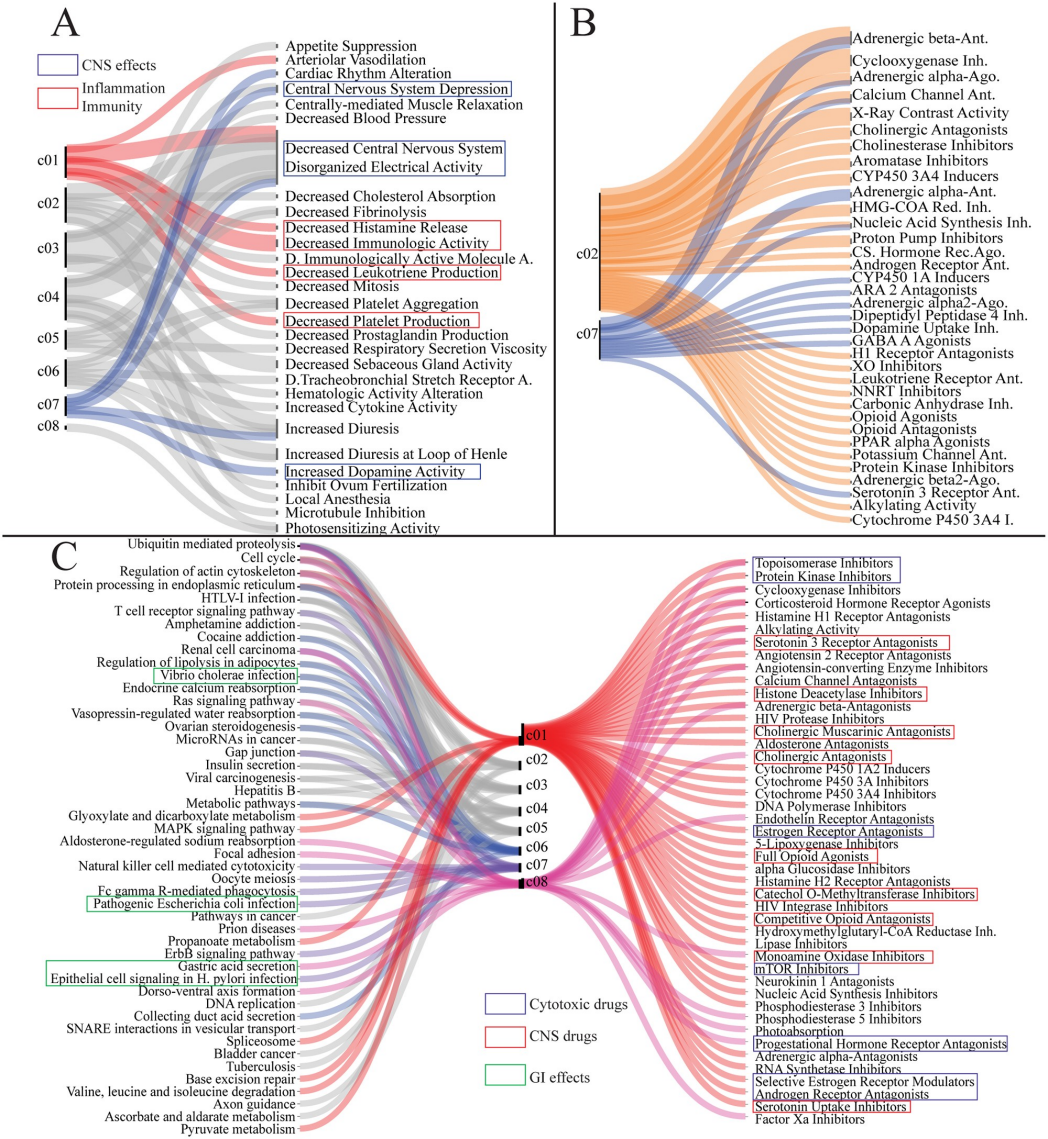
Cluster annotation using FDA NDCD and KEGG databases. A-Bipartite graph of the drug clusters and FDA NDCD PE linking each cluster to reported physiological effects of drugs. B-Bipartite graph of the drug clusters and FDA NDCD EPC linking the clusters to the reported pharmacological class. C-Graph linking the drug clusters to the FDA NDCD MoA and KEGG-enriched pathways of the gene pertaining to each cluster. The graph links the drug mechanism of action to the pathways perturbed by the drug.

The construction of the drug feature matrix consisting of gene and metabolic reaction vectors per drug could facilitate the use of clustering techniques to classify drugs in the gene and metabolism space. In particular, drugs that have similar gene expression and metabolic profiles could be suggested for repurposing in novel indications. Using Jaccard-Louvain [48] the community detection algorithm, we identified eight drug clusters based on their genetic and metabolic signatures (Fig 4-A). Each cluster had a stability and purity index greater than 0.75 (S10-D Fig), thereby validating the obtained clusters. In particular, transcriptional and intestinal metabolic activities were aligned with the identified clusters (Fig 4-B), such as each drug cluster could have either high or low metabolic and transcriptomic activity. Interestingly, the identified clusters did not map to the FDA NDCD’s Established Pharmacological Class (EPC) (S10-A Fig) suggesting that classical indication-based classification may overlook the genetic and molecular aspects of small molecule pharmacodynamics. Most small molecules had a low genetic and metabolic fingerprint and mainly targeted the various transport subsystems (S10-C Fig) of the enterocytes, which is consistent with transport being a chief function of the gut wall. Clusters one and eight involved a high number of genes and metabolic reactions mainly due to cytotoxic drugs, which were also reflected by the presence of terms linked to inflammation and immunity in the bipartite graph linking the drugs to the FDA NDCD’s Physiological Effects (PEs) (Fig 5-A). Additionally, clusters eight and one were linked to malignant side effects (S10-A Fig).

Cluster seven had a high number of active fluxes in the small intestine epithelial cells. Interestingly, a number of terms linked to the central nervous system were found (Fig 5-A), which hints to potential gut-brain shared molecular processes, probably linked to the similar composition of the blood-brain barrier and the gut wall transporters [58]. In addition to cluster seven, cluster two had a low transcriptomic and a high metabolic profile. The EPCs linked to these clusters were mostly compounds whose action is mediated through metabolic functions, such as xanthine oxidase, or signaling, such as PPAR*α* molecule binding in the bipartite graph linking the drugs to their EPCs (Fig 5-B). Ubiquitous targets, including cyclooxygenase and histamine receptors, could consequentially induce pronounced metabolic effects.

In the high transcription and high metabolism profiles represented by cluster one and eight, the presence of molecules acting on the central nervous system by their FDA NDCD’s Mechanism of Action (MoA) (Fig 5-C), confirmed links between the gut and brain metabolisms. Additionally, since clusters one and eight encompassed anticancer drugs, as we previously observed, this finding further supports ongoing repurposing trials of antidepressants in cancer therapy [59]. Moreover, neurokinin-1 antagonists, a class of drugs prescribed for the suppression of cytotoxic drug-induced emesis, and 5-lipoxygenase inhibitors, indicated for inflammatory bowel disease, had a high genetic and metabolic profile indicating potential links between gut symptomatology and genome-wide transcriptional and metabolic modulation. We further enriched the top differentially enriched genes in each cluster in the KEGG database [54, 56] (Fig 5-C) and selected the terms pertaining to gastrointestinal physiology. Epithelial cell signalling in *Helicobacter pylori* infection was linked to cluster seven, which had a low transcriptomic and high metabolic profile suggesting metabolism-modulated signaling through kinases following the infection. The *Escherichia coli* infection term belonged to this cluster, which suggests that both pathogens might involve the same kinase but also that similar treatment strategies may be able to combat their infections. Phenotypes involving signaling mechanisms rather than metabolism, e.g., *Vibrio cholerae* infection, belonged to cluster six that had a low intestinal metabolic fingerprint.

Taken together, the multilayer biology of drug effects could accurately predict iatrogenic gastrointestinal effects using an SVM classifier. The clustering of drugs based on their metabolic and genetic signature has the potential to unravel potential similarities in the model of action of compounds in relation to their physiological effects.

## Discussion

The prediction of side effects using only *in vitro* data of small molecules is a requisite of safe first-in-human trials and low attrition rates in the clinical phases. The connectivity map of drug signatures [6] has provided a large set of gene expression profiles corresponding to small-molecules perturbagens. Here, we modeled the metabolism of sIECs under drug-induced perturbations to predict iatrogenic effects using a machine learning classifier. The sampling of the distribution of reaction rates of the drug-specific sIEC metabolic models taken as features provided better classification results than using the extremal reaction flux values obtained using FVA. Moreover, combining gene expression with modeling captured both metabolic and nonmetabolic effects in relation to side effect development. Particularly, the addition of the modeling of the metabolism of the sIEC to the gene expression data could capture local gut metabolism and improve the detection of side effects. Finally, clustering the drugs with respect to their metabolic and transcriptomic fingerprints, as opposed to their chemical structure and pharmacological class, suggested drug repurposing strategies, which could yield new therapeutic alternatives.

### Model generation and parameter selection

The connectivity map [6] has provided a large-scale resource of small-molecule transcriptomic signatures and enabled the genome-wide assessment of drug off-target effects, thereby expanding pharmacology beyond the study of the drug primary target alone. The integration of drug-induced gene expression with generic metabolic models of human metabolism could be used to identify key disrupted metabolic functions [10] resulting from adverse reactions. Similarly, the integration of known target effects of drugs as identified from DrugBank [60] and flux bounds obtained by FVA as features [27] has been used to predict accurately several labels of side effects. Although, this approach remains limited to drugs with inhibitory effects on metabolic targets and *a fortiori* of known targets. In our approach, the integration of drug-induced gene expression with metabolic networks allowed to circumvent the inhibitory target limitation [61] and extend to other classes of drugs. Additionally, the integration of gene expression allowed the modeling of drug off-target effects, which were suggested to be the main driver of side effects. We showed that informing the classifier with the distribution of metabolic fluxes per reaction using sampling rather than by providing the bounds of the reaction using FVA increased the predictive power of the classifier (Fig 2-A and 2-B). Additionally, restricting the predictions to a set of organ-specific side effects using a manually curated tissue-specific metabolic model captured the local metabolism in relation to the emergence of organ-specific adverse reactions. This finding was in accordance with a recent review [62] that demonstrated a link between organ-specific functions of drug targets and the likelihood of organ-specific side effects.

Improving the prediction of side effects relies greatly on the quality and completeness of the dataset used. Weighing the variables by the side effect frequencies per drug likely improves the predictions and can leverage the prediction of rare side effects. Nevertheless, only 46% of side effects had associated frequency information whose inclusion did not improve the prediction accuracy (S7 Fig). The missing information could be potentially filled by either manual expert curation or crossing databases. Moreover, the PEs and mechanisms of action in the FDA NDCD were missing for many drugs as well. Additionally, the chronopharmacology of drug action has been found to be also of importance in detecting the emergence of side effects [63]. The connectivity map provides many experiments at several time intervals that we did not exploit in our analysis because not all drug-induced gene expression have been measured at different time points. Such data could transform predictions from snapshots of transcription and metabolism to dynamical models linking the emergence of side effects to time-dependent processes.

### Sampling the metabolic model of the gut wall achieved the highest prediction accuracy

Conceptually, drug-induced gene expression could play a significant role in the genesis of adverse reactions and have been predictive towards side effects classification, especially when combined with other drug features, such as chemical structure and cell morphology after treatment [8]. The combination of local gastrointestinal metabolism constrained by metabolic gene expression and the differential expression of nonmetabolic genes achieved the most accurate prediction of gastrointestinal side effects blackas assessed by the micro-average (Fig 2-A) where each class is treated equally, and the micro-average (Fig 2-B) with a correction for the class imbalance. The improvement in the multilabel classification was related to an increase in the AUROC of a specific set of side effects (Fig 3) but not all side effects had an improved prediction using combined gene expression and metabolism (S3 Table). This set consisted of nine side effects, including gastrointestinal pain, disorder, obstruction, and fistula. The reasons for such a decrease are related to our parameter optimization procedure. In fact, we performed a class-wide optimization of the classifier parameters such as the number of features and the feature selection algorithm. A class-specific optimization procedure could be tested in future applications but would i) require larger computational resrouces, ii) increase the complexity of the classifier, and iii) reduce the interpretation of common factors related to all the gastrointetsinal side effects as our approach optimizes for all the side effects simulatenously.

The combination of multiple layers of biology consisting of transcriptomic and predicted metabolic reaction fluxes as features was pivotal to capturing drug-induced perturbations related to side effects. Furthermore, the approach could be scaled to several tissues to include all the labels of side effects using manually curated models of human metabolism [64]. Remarkably, sampling metabolic models alone achieved good accuracy taking into account that only the metabolic subset of genes from the connectivity map was modeled. In particular, the AUROC equaled 0.931 and was not statistically different (p = 0.29) from classifiers using gene expression as features (AUROC = 0.935). Therefore, we suggest that a reduced set of *in vitro* experiments to measure the differential expression of metabolic genes would give an invaluable insight into the emergence of adverse reactions of a new chemical entity in the preclinical phase, which could guide the rational design of first-in-human trials. Furthermore, the emergence of whole-cell models [65, 66], which integrate metabolism alongside with several physiological functions, could be used to map nonmetabolic genes onto computational models of the cell to capture the cell-wide disruption of physiological processes leading to the emergence of side effects. With the generated combined gene expression and sIEC metabolic reactions matrix in hand, we classified the small molecules with respect to their signatures to highlight their shared features.

### Drug reclassification beyond the chemical class

Drugs are often classified based on their pharmacological indications and their chemical family. The many examples of marketed drugs repurposed for new indications [67] show that a small molecule can indeed have many, diverse effects. Drug repurposing has gained great interest in recent years because it can significantly accelerate the drug development process using compounds with well-documented safety. Additionally, transcriptome-based clustering could be used to recommend new activities for investigational molecules [68]. To find shared properties of drugs, we identified clusters of compounds that share similar genetic and metabolic signatures in the gut wall (Fig 4-A and 4-B). Interestingly, compounds that involved a high number of metabolic reactions with a high amplitude of variation included CNS drugs, such as serotonin antagonists, which were indicated primarily for psychotic episodes and were later suggested to treat chemotherapy-induced emesis (Fig 4-C). Moreover, these compounds belonged to the same cluster as neurokinin inhibitors, which are indicated for the prevention of emesis (Fig 4-E). Serotonin antagonists are also indicated to treat inflammatory bowel syndrome, which further showed a similarity between the blood-brain barrier and the gut wall metabolism and gene expression (Fig 4-E). Furthermore, anticancer drugs and the drugs that treat their side effects, the antiemesis drugs, clustered together in the high transcriptomic, high metabolic activity cluster, further supporting the idea that reversing the molecular fingerprint of a compound could reverse its effects. Particularly, reversing the fingerprint of the compound locally in the gut wall would be a potential strategy to reverse gastrointestinal side effects of drugs through the administration of codrugs, while preserving its primary activity in the target tissue. Interestingly, the clusters of drugs that we identified in our analysis did not match FDA marketing date (S10-B Fig). Despite the emergence of the key-lock paradigm [69] in drug development using molecular dynamics and docking experiments in the early 1990s that decreased the number of drugs interacting with a high number of targets, colloquially called ‘dirty drugs’, there seems to remain opportunities to further enhance the design of precise therapies.

In conclusion, we developed and employed a multilabel support vector machine on the genetic and metabolic fingerprints of marketed small-molecule compounds to accurately predict the occurrence of gastrointestinal side effects. The drug features could be used to classify drugs based on their metabolic and genetic profiles, which is a promising avenue for drug repurposing to reverse side effects and unravel new indications. The development of large-scale, publicly available compound resources combined with complex mathematical models of cellular biology may represent a new method of providing patients with safer and more efficient therapies.

## Supporting information

S1 Fig Comparison of multilabel classifiers. Comparison of multilabel classifiers. Comparison of multilabel classifiers. Four classifiers, namely, logistic regression, Naïve Bayes, random forest, and support vector machine, were compared in their predictive capabilities measured by the F1-score, accuracy, label weighted accuracy, label weighted recall, AUROC, and AUPR.
(TIF)

S2 Fig Feature selection algorithm comparison. Comparison of 11 feature selection algorithms with respect to the AUROC of individual intestinal side effects with the 95% confidence interval for the mean in red and one standard deviation in blue.
(TIF)

S3 Fig ReliefF’s k value comparison. Comparison of k values for the feature selection algorithm ReliefF through the AUROC of classifiers of individual side effects with the 95% confidence interval for the mean in red and one standard deviation in blue. The highest mean (0.83) was achieved for k = 80.
(TIF)

S4 Fig Comparison of the number of selected features. Comparison of the effect of the number of the most predictive features in the classification performance as assessed by the AUROC.
(TIF)

S5 Fig Assessment of the cross-validation loss. Comparison of cross-validation methods on the loss calculated as the number of misclassified side effects per drug over the total number of side effects, and the predictability of the individual side effects as reflected by the AUROC. Outliers in the loss are rare side effects that have a small number of data points. The 3-fold cross-validation ensured a lower loss and highest AUROC for out-of-sample drugs. Left: distribution of the AUROC of individual side effects with the 95% confidence interval for the mean in red and one standard deviation in blue. Right: boxplot of the loss calculated for each cross-validation method.
(TIF)

S6 Fig Effect of class balance. Comparison of the effects of the class balance set as the misclassification cost on the outcome of the classification as determined by the AUROC curve. The misclassification cost, set to the inverse of label frequencies, could be used to obtain a mean of 0.875 of the AUROC of the individual intestinal side effects as opposed to 0.86 without class balance.
(TIF)

S7 Fig Effect of observation weight. Comparison of the effect of adding observation weights to the classifier compared to the AUROC. The weights of drugs per label were set to their frequencies reported in SIDER. Weighing observations had a mean area under the curve of 0.830 while unweighted observations had a mean of 0.836.
(TIF)

S8 Fig Comparison of SVM kernel functions. Comparison of SVM kernel functions as a function of the AUROC curve of individual side effects. Overall, the Gaussian kernel had the highest predictive capabilities.
(TIF)

S9 Fig Automatic tuning of kernel parameters. Effect of automatic and manual hyperparameter optimization with respect to 20% holdout accuracy as an objective function. The manually obtained parameters could be used to obtain a higher predictive capability of the classifier as measured by the individual side effect AUROC curve.
(TIF)

S10 Fig Drug cluster validation and characteristics. Drug cluster validation and characteristics. A-Graph linking drug clusters, intestinal side effects, and FDA NDCD’s EPC. B-Bipartite graph of drug clusters and the corresponding FDA NDCD’s reported marketing date. C-Bipartite graph of drug clusters and enriched metabolic and transport subsystems. The flow chart was created using Rawgraphs [53]. D-Cluster stability and purity provided a means for cluster validation.
(TIF)

S1 Table Optimal classifier parameters. (PDF)

S2 Table Automatically optimized SVM hyperparameters. (PDF)

S3 Table AUROC of the predicted side effect. AUROC curve of the predicted side effect using a multilabel support vector machine classifier with combined gene expression and sampled metabolic flux as features.
(PDF)
