# Supplementary figures and images for: Predicting gastrointestinal drug effects using contextualized metabolic models
Source: PLoS Comput Biol. 2019 Jun 26;15(6):e1007100. doi: 10.1371/journal.pcbi.1007100 (PMC6594586; doi:10.1371/journal.pcbi.1007100)

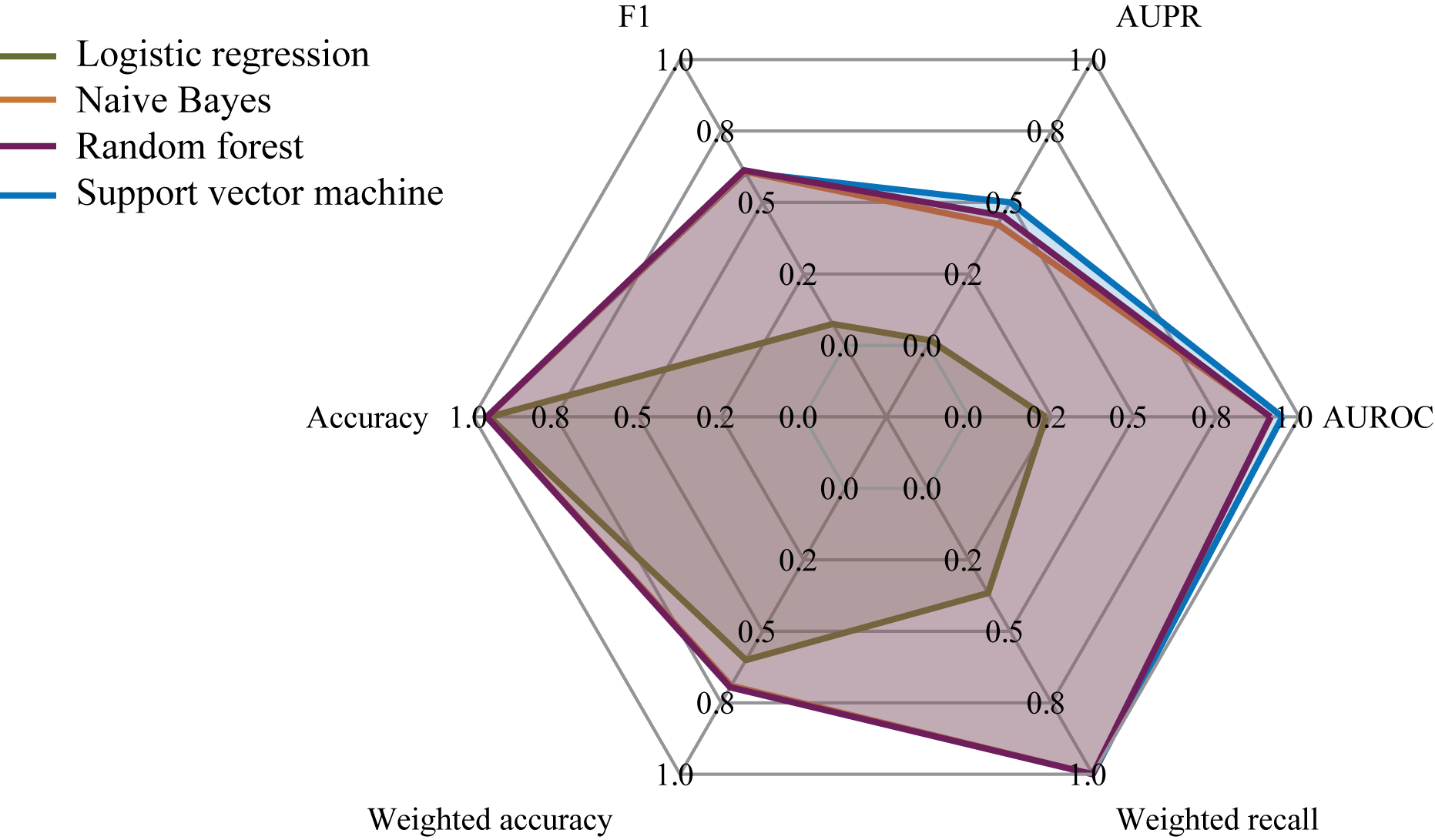

Supplement: S1 Fig — Comparison of multilabel classifiers. Comparison of multilabel classifiers. Four classifiers, namely, logistic regression, Naïve Bayes, random forest, and support vector machine, were compared in their predictive capabilities measured by the F1-score, accuracy, label weighted accuracy, label weighted recall, AUROC, and AUPR. (TIF) [file pcbi.1007100.s001.tif]

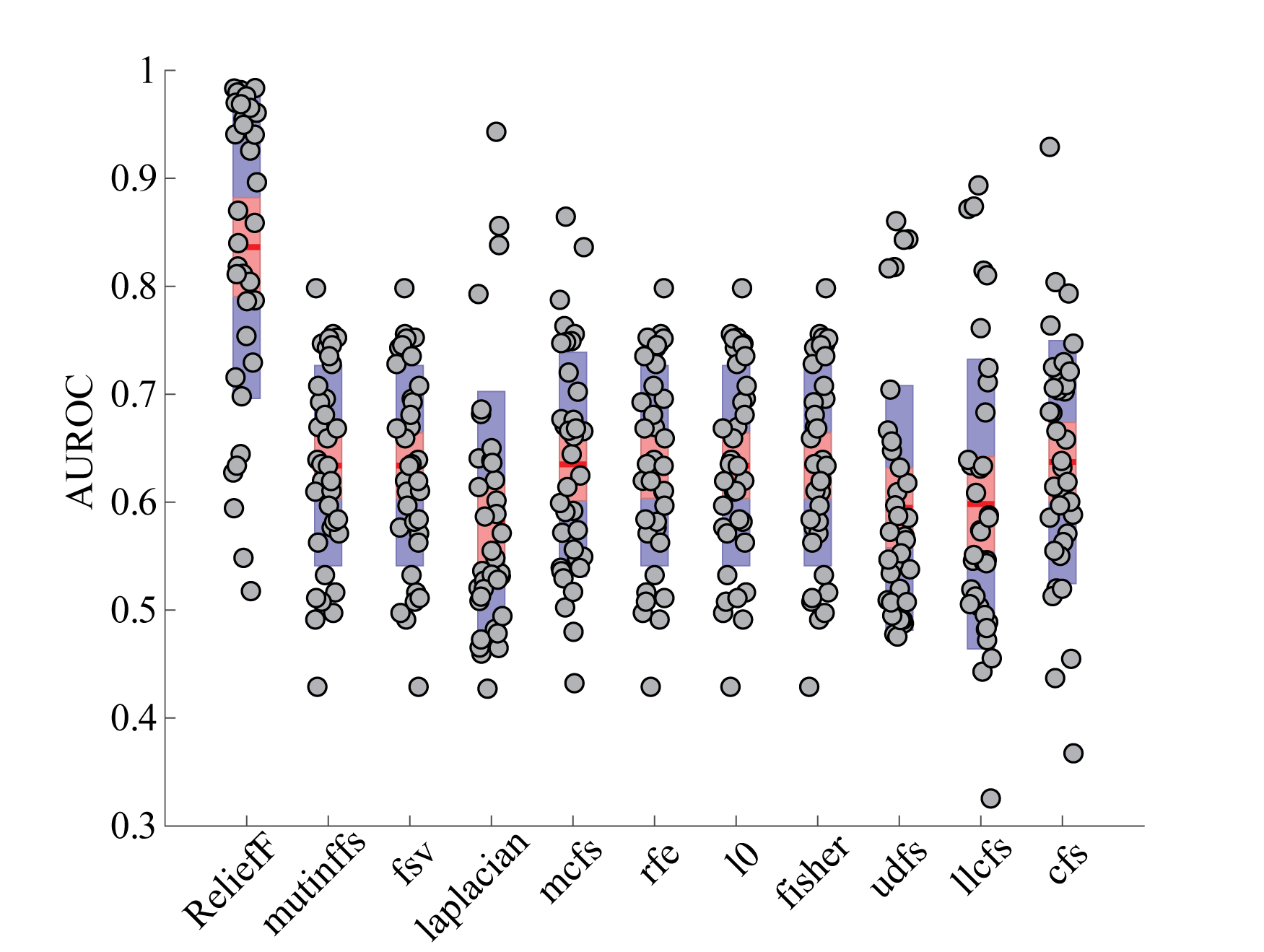

Supplement: S2 Fig — Comparison of 11 feature selection algorithms with respect to the AUROC of individual intestinal side effects with the 95% confidence interval for the mean in red and one standard deviation in blue. (TIF) [file pcbi.1007100.s002.tif]

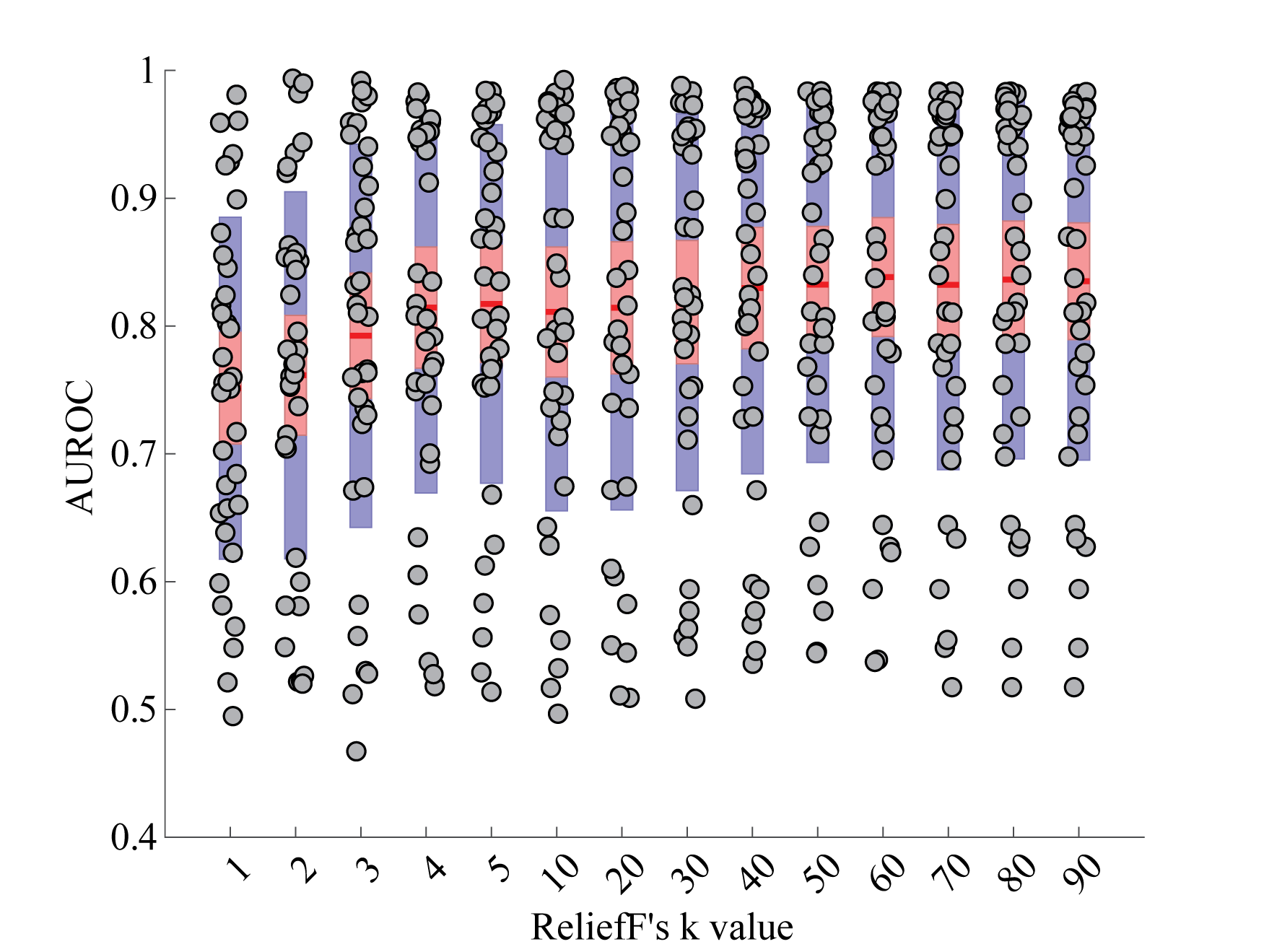

Supplement: S3 Fig — Comparison of k values for the feature selection algorithm ReliefF through the AUROC of classifiers of individual side effects with the 95% confidence interval for the mean in red and one standard deviation in blue. The highest mean (0.83) was achieved for k = 80. (TIF) [file pcbi.1007100.s003.tif]

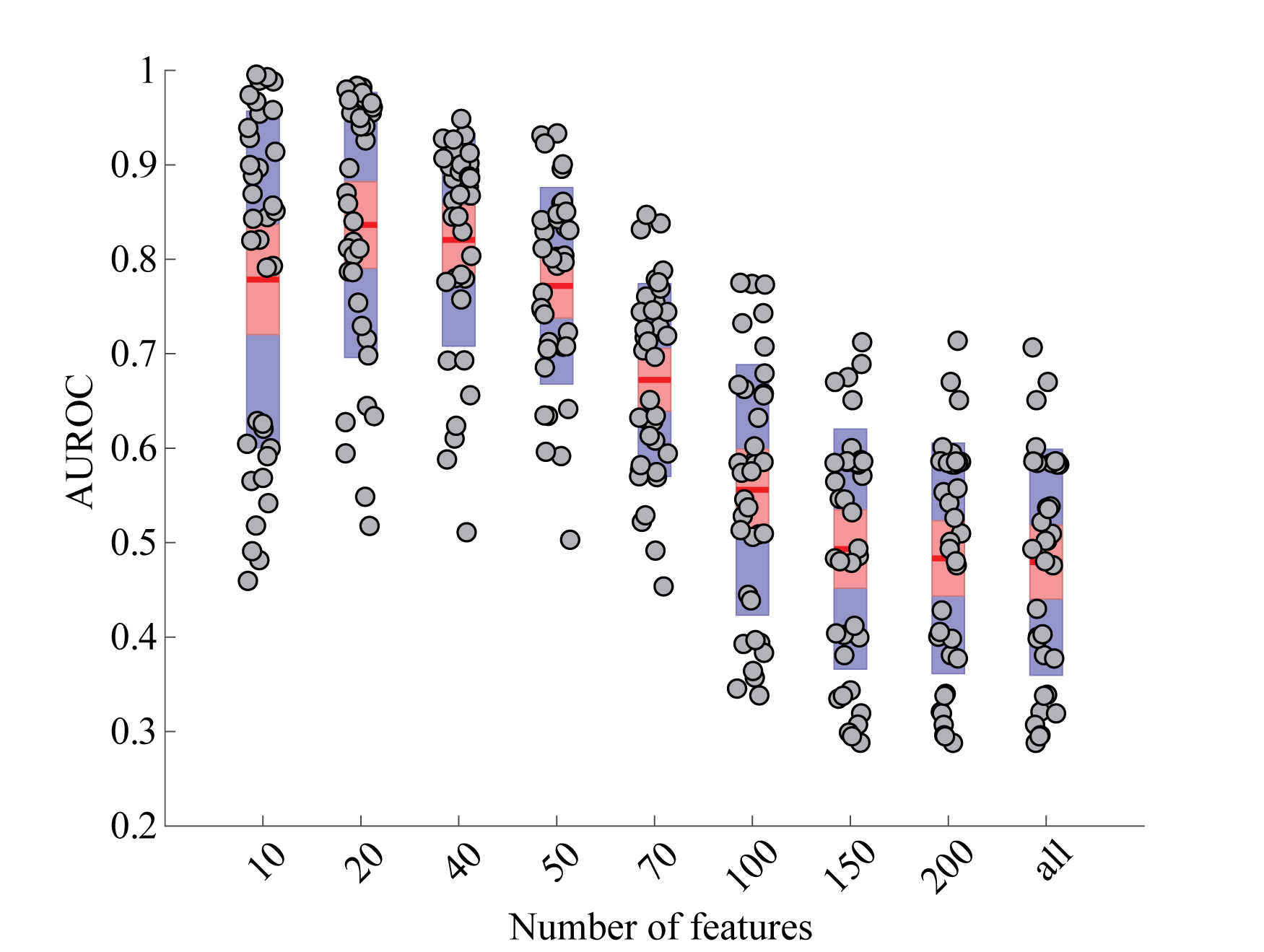

Supplement: S4 Fig — Comparison of the effect of the number of the most predictive features in the classification performance as assessed by the AUROC. (TIF) [file pcbi.1007100.s004.tif]

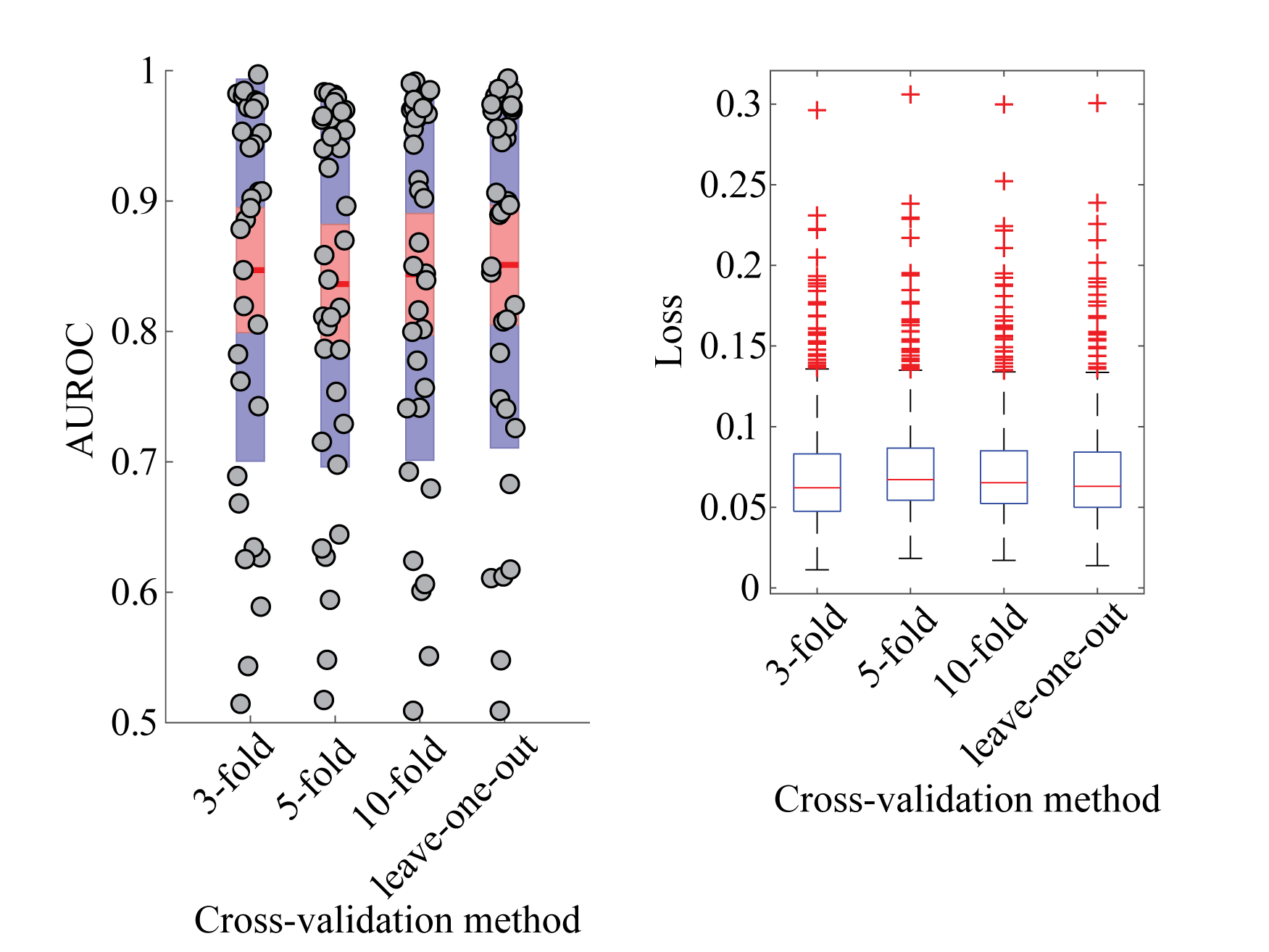

Supplement: S5 Fig — Comparison of cross-validation methods on the loss calculated as the number of misclassified side effects per drug over the total number of side effects, and the predictability of the individual side effects as reflected by the AUROC. Outliers in the loss are rare side effects that have a small number of data points. The 3-fold cross-validation ensured a lower loss and highest AUROC for out-of-sample drugs. Left: distribution of the AUROC of individual side effects with the 95% confidence interval for the mean in red and one standard deviation in blue. Right: boxplot of the loss calculated for each cross-validation method. (TIF) [file pcbi.1007100.s005.tif]

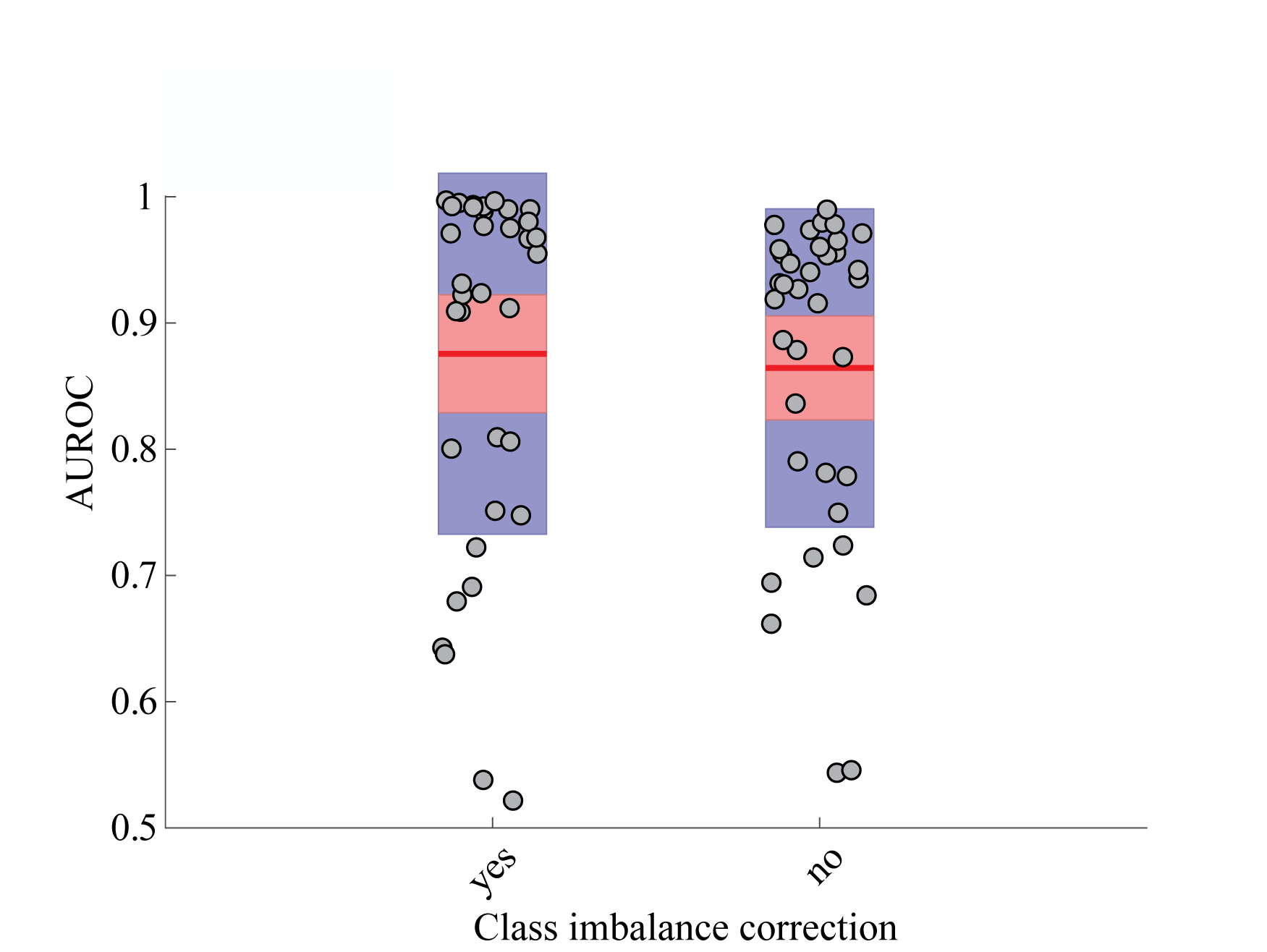

Supplement: S6 Fig — Comparison of the effects of the class balance set as the misclassification cost on the outcome of the classification as determined by the AUROC curve. The misclassification cost, set to the inverse of label frequencies, could be used to obtain a mean of 0.875 of the AUROC of the individual intestinal side effects as opposed to 0.86 without class balance. (TIF) [file pcbi.1007100.s006.tif]

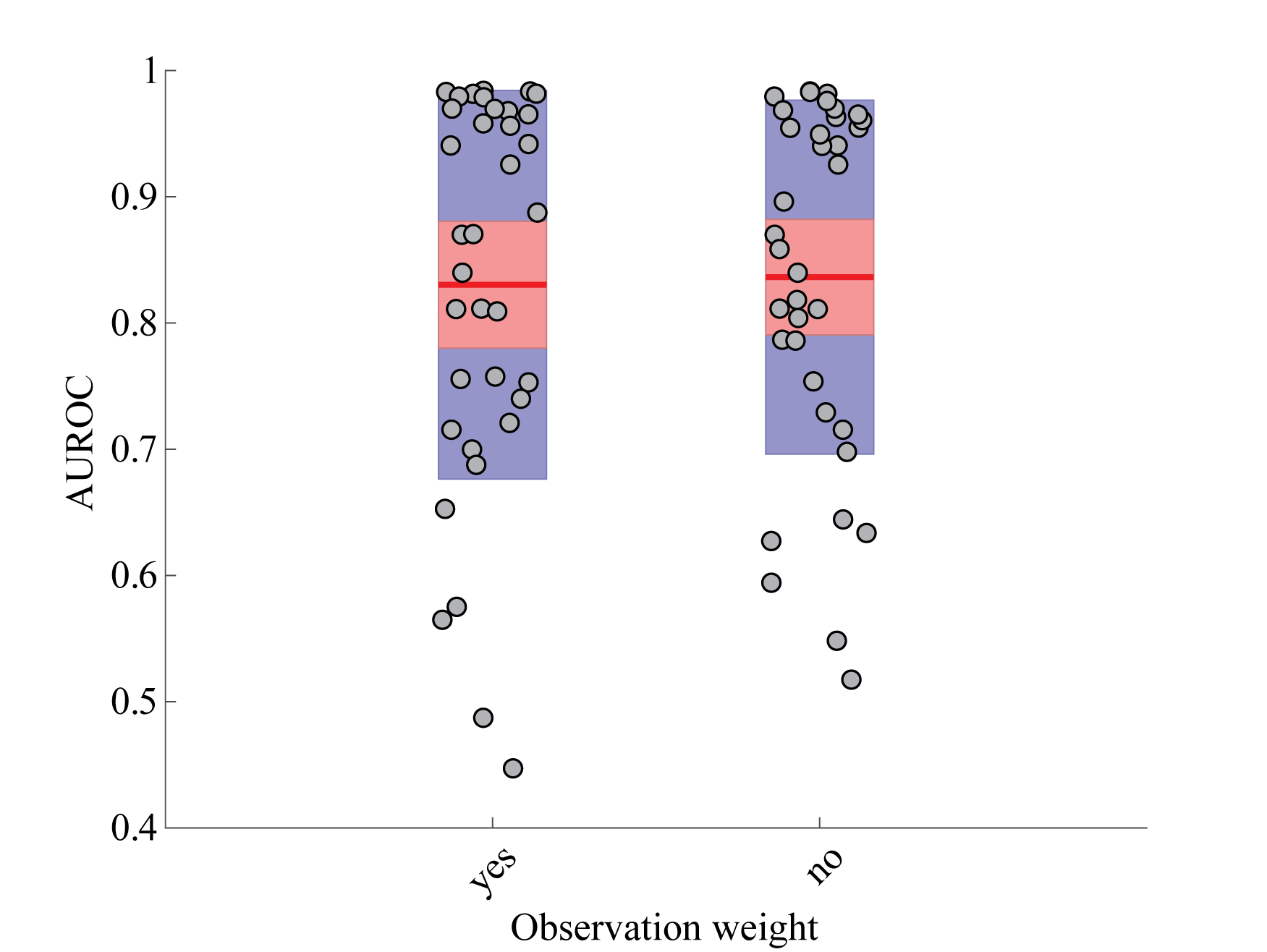

Supplement: S7 Fig — Comparison of the effect of adding observation weights to the classifier compared to the AUROC. The weights of drugs per label were set to their frequencies reported in SIDER. Weighing observations had a mean area under the curve of 0.830 while unweighted observations had a mean of 0.836. (TIF) [file pcbi.1007100.s007.tif]

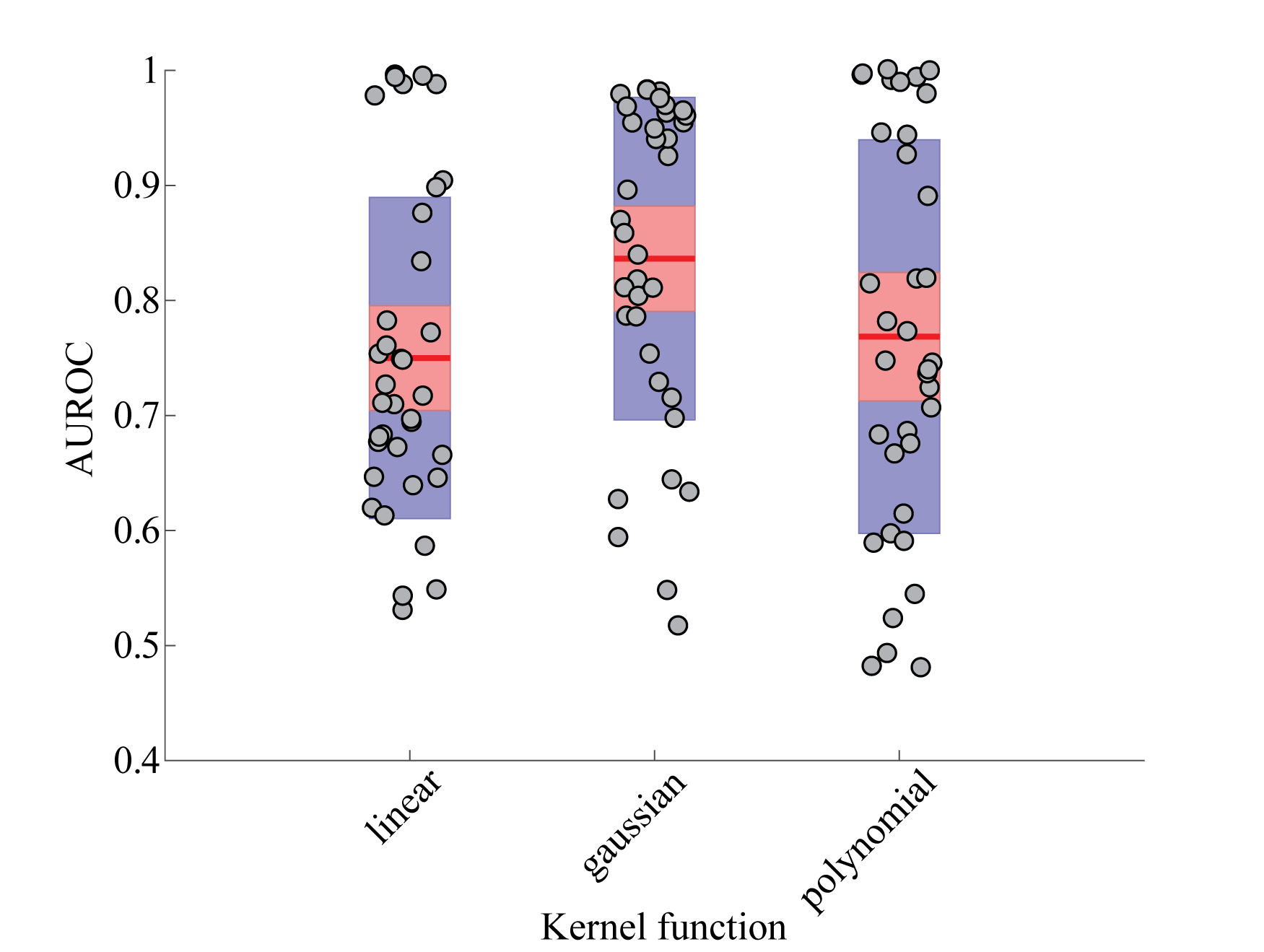

Supplement: S8 Fig — Comparison of SVM kernel functions as a function of the AUROC curve of individual side effects. Overall, the Gaussian kernel had the highest predictive capabilities. (TIF) [file pcbi.1007100.s008.tif]

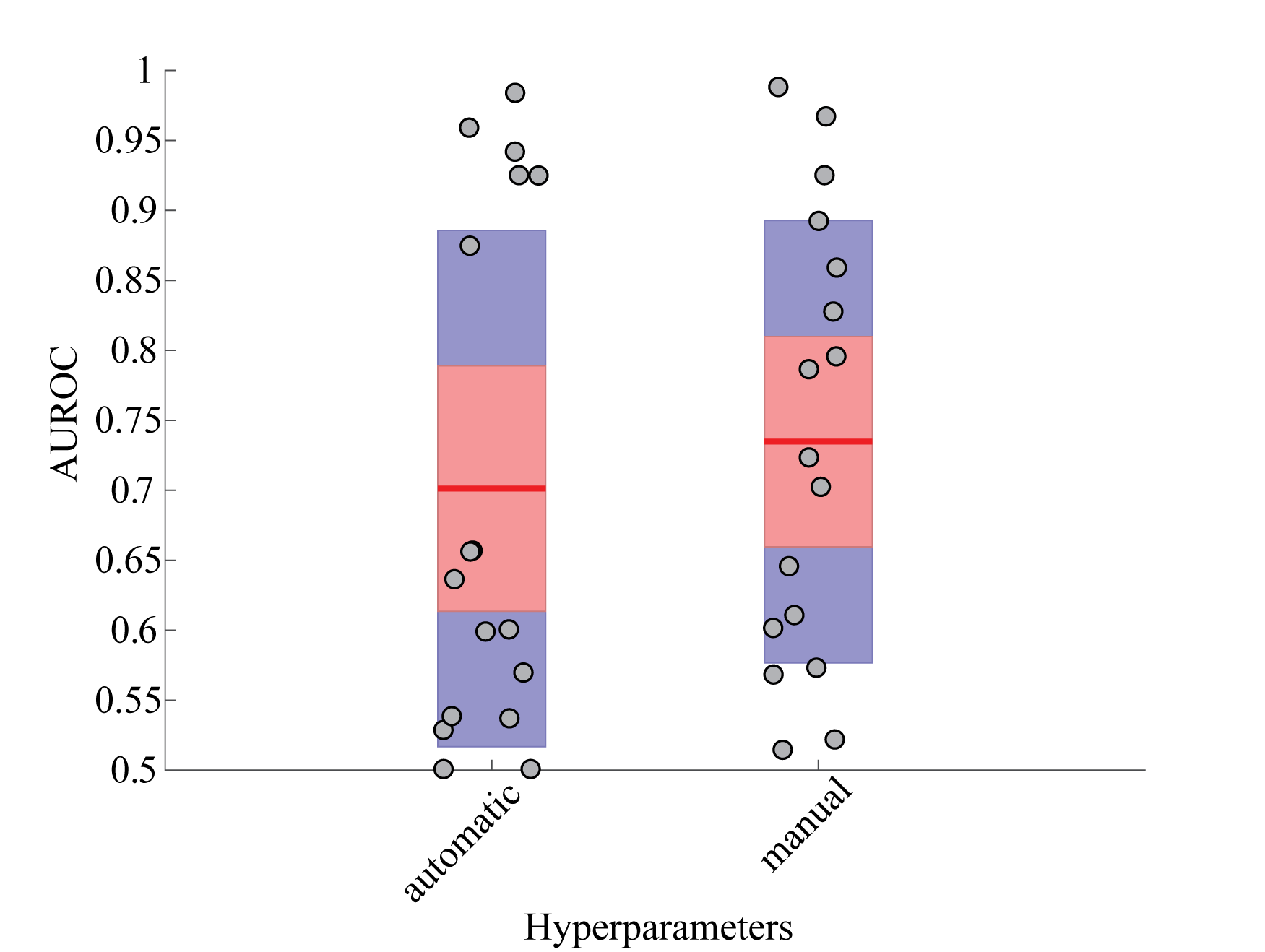

Supplement: S9 Fig — Effect of automatic and manual hyperparameter optimization with respect to 20% holdout accuracy as an objective function. The manually obtained parameters could be used to obtain a higher predictive capability of the classifier as measured by the individual side effect AUROC curve. (TIF) [file pcbi.1007100.s009.tif]

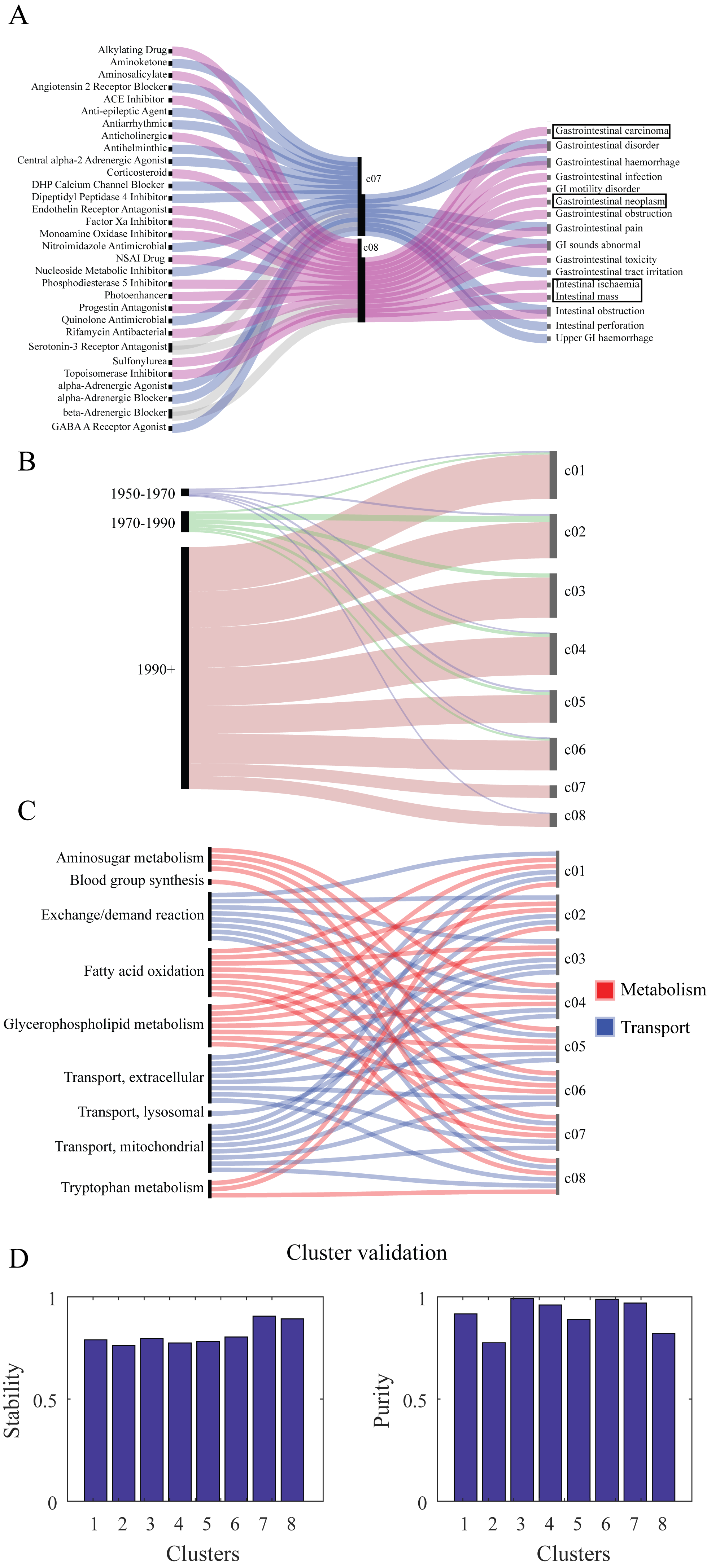

Supplement: S10 Fig — Drug cluster validation and characteristics. A-Graph linking drug clusters, intestinal side effects, and FDA NDCD’s EPC. B-Bipartite graph of drug clusters and the corresponding FDA NDCD’s reported marketing date. C-Bipartite graph of drug clusters and enriched metabolic and transport subsystems. The flow chart was created using Rawgraphs [53]. D-Cluster stability and purity provided a means for cluster validation. (TIF) [file pcbi.1007100.s010.tif]
