# Supplementary material for: Predicting gastrointestinal drug effects using contextualized metabolic models
Source: PLoS Comput Biol. 2019 Jun 26;15(6):e1007100. doi: 10.1371/journal.pcbi.1007100 (PMC6594586; doi:10.1371/journal.pcbi.1007100)
Supplement: S1 Table — (PDF) [file pcbi.1007100.s011.pdf]

Table S1: Support vector machine parameter summary

Parameter	Optimal value/algorithm
Feature selection algorithm	ReliefF
k-ReliefF	80
Number of features	20
Cross-validation	3-fold cross-validation
Class balance	Inverse of class frequencies
Observation weights	Drug side effect frequency per drug
SVM kernel	Gaussian
