# Supplementary material for: Predicting gastrointestinal drug effects using contextualized metabolic models
Source: PLoS Comput Biol. 2019 Jun 26;15(6):e1007100. doi: 10.1371/journal.pcbi.1007100 (PMC6594586; doi:10.1371/journal.pcbi.1007100)
Supplement: S2 Table — (PDF) [file pcbi.1007100.s012.pdf]

Table S2: Automatically optimized SVM hyperparameters.

Hyperparameter	Value/Range
Standardize data	True,false
Kernel	Linear, Gaussian, polynomial
Polynomial order	[2,5]
Box constraints	[1e-6,1e+4]
Kernel scale	True,false
