# Supplementary material for: Predicting gastrointestinal drug effects using contextualized metabolic models
Source: PLoS Comput Biol. 2019 Jun 26;15(6):e1007100. doi: 10.1371/journal.pcbi.1007100 (PMC6594586; doi:10.1371/journal.pcbi.1007100)
Supplement: S3 Table — AUROC curve of the predicted side effect using a multilabel support vector machine classifier with combined gene expression and sampled metabolic flux as features. (PDF) [file pcbi.1007100.s013.pdf]

Table S3: Area under the ROC curve of the predicted side effect using a multi-label support vector machine classifier with combined gene expression and sampled metabolic flux as features. GM is combined gene expression and sampled metabolism, G is gene expression, M is sampled metabolic, M-FVA is metabolism assessed by reaction bounds using FVA.

Side effect	GM	G	M	M-FVA
Gastrointestinal pain	0.52	0.6	0.52	0.51
Gastrointestinal disorder	0.53	0.64	0.51	0.55
Functional gastrointestinal disorder	0.63	0.71	0.74	0.63
Gastrointestinal haemorrhage	0.64	0.62	0.56	0.47
Intestinal obstruction	0.67	0.6	0.53	0.51
Intestinal perforation	0.69	0.68	0.49	0.67
Gastrointestinal tract irritation	0.72	0.7	0.59	0.45
Gastrointestinal perforation	0.74	0.71	0.72	0.58
Gastrointestinal obstruction	0.75	0.98	0.72	0.78
Gastrointestinal fistula	0.79	0.98	0.94	0.94
Intestinal ulcer	0.8	0.76	0.48	0.26
Gastrointestinal hypomotility	0.8	0.92	0.72	0.78
Gastrointestinal sounds abnormal	0.9	0.78	0.72	0.71
Gastrointestinal ulcer haemorrhage	0.9	0.94	0.67	0.91
Gastrointestinal infection	0.91	0.9	0.64	0.58
Gastrointestinal necrosis	0.92	0.99	0.76	0.71
Gastrointestinal toxicity	0.92	0.71	0.66	0.56
Upper gastrointestinal haemorrhage	0.93	0.81	0.72	0.45
Lower gastrointestinal haemorrhage	0.95	0.94	0.84	0.36
Gastrointestinal carcinoma	0.96	0.7	0.8	0.6
Gastrointestinal ulcer perforation	0.96	0.99	0.99	1.0
Gastrointestinal motility disorder	0.97	0.95	0.58	0.5
Intestinal infarction	0.97	0.95	0.91	0.49
Gastrointestinal ulcer	0.97	0.95	0.87	0.96
Gastrointestinal stromal tumour	0.97	0.93	0.88	0.64
Pneumatosis intestinalis	0.97	0.86	0.98	0.8
Intestinal ischaemia	0.98	0.94	0.98	0.98
Large intestine polyp	0.98	0.82	0.78	0.64
Gastrointestinal malformation	0.98	0.99	0.9	0.49
Gastrointestinal candidiasis	0.99	0.97	0.84	0.49
Benign gastrointestinal neoplasm	0.99	0.78	0.7	0.68
Gastrointestinal inflammation	0.99	0.99	0.94	0.63
Diverticulum intestinal	0.99	0.98	0.98	0.99
Large intestine perforation	0.99	0.96	0.97	0.99
Small intestinal obstruction	0.99	0.74	0.99	0.91
Intestinal haemorrhage	0.99	0.95	0.87	0.59
